# Increased biomass and lipid production by continuous cultivation of *Nannochloropsis salina* transformant overexpressing a bHLH transcription factor

**DOI:** 10.1002/bit.26894

**Published:** 2019-01-03

**Authors:** Nam Kyu Kang, Eun Kyung Kim, Min‐Gyu Sung, Young Uk Kim, Byeong‐ryool Jeong, Yong Keun Chang

**Affiliations:** ^1^ Advanced Biomass R&D Center, Yuseong‐gu Daejeon Republic of Korea; ^2^ Department of Chemical and Biomolecular Engineering, KAIST, Yuseong‐gu Daejeon Republic of Korea

**Keywords:** bHLH transcription factor, biofuels, continuous cultivation, microalgae, nannochloropsis salina

## Abstract

Microalgae are promising feedstocks for sustainable and eco‐friendly production of biomaterials, which can be improved by genetic engineering. It is also necessary to optimize the processes to produce biomaterials from engineered microalgae. We previously reported that genetic improvements of an industrial microalga *Nannochloropsis salina* by overexpressing a basic helix‐loop‐helix transcription factor (NsbHLH2). These transformants showed an improved growth and lipid production particularly during the early phase of culture under batch culture. However, they had faster uptake of nutrients, resulting in earlier starvation and reduced growth during the later stages. We attempted to optimize the growth and lipid production by growing one of the transformants in continuous culture with variable dilution rate and feed nitrogen concentration. Relative to wild‐type, NsbHLH2 transformant consumed more nitrate at a high dilution rate (0.5 day ^−1^), and had greater biomass production. Subsequently, nitrogen limitation at continuous cultivation led to an increased fatty acid methyl ester production by 83.6 mg l ^−1^ day ^−1^. To elucidate genetic mechanisms, we identified the genes containing E‐boxes, known as binding sites for bHLH transcription factors. Among these, we selected 18 genes involved in the growth and lipid metabolism, and revealed their positive contribution to the phenotypes via quantitative real‐time polymerase chain reaction. These results provide proof‐of‐concept that NsbHLH2 can be used to produce biomass and lipids.

## 1 INTRODUCTION

The high dependence on petroleum‐based energy sources has led to an accumulation of greenhouse gases and other environmental problems (Demirbas, 2017). Therefore, there is a growing interest in the development of renewable and sustainable energy sources such as biomass. Microalgae are promising energy sources because they have a higher biomass and lipid productivity than the crop plants (Ho, Ye, Hasunuma, Chang, & Kondo, 2014; Parmar, Singh, Pandey, Gnansounou, & Madamwar, 2011; Williams & Laurens, 2010).

Despite their advantages, biofuel production from microalgae has practical limitation about economic feasibility. Genetic engineering could resolve this problem by developing microalgae that have increased lipid and biomass productivity (Radakovits, Jinkerson, Darzins, & Posewitz, 2010). However, it is difficult to engineer microalgae with desirable phenotypes by the manipulation of individual enzymes. Thus, transcription factor (TF) engineering which regulates the expression of various genes simultaneously emerging in microalgae fields (Bajhaiya, Ziehe Moreira, & Pittman, 2017; Courchesne, Parisien, Wang, & Lan, 2009). It has been reported that nitrogen‐responsive regulator and phosphorous starvation response 1 (PSR1) TFs play important roles in lipid accumulation (Bajhaiya, Dean, Zeef, Webster, & Pittman, 2016; Boyle et al., 2012). Other studies reported an increased lipid content in *Chlamydomonas* and *Chlorella* cells that overexpressed DNA binding with one finger (Dof)‐type TFs (Ibanez‐Salazar et al., 2014; Zhang et al., 2014). In addition, we reported that the biomass and lipid production could be improved by the overexpression of stress‐ and/or lipid‐related TFs, including *Nannochloropsis salina* by overexpressing a basic helix‐loop‐helix transcription factor (NsbHLH2), AtWRI1, and NsbZIP1 (Kang et al., 2015a; Kang et al., 2017; Kwon et al., 2018).

Various cultivation methods are used for microalgal lipid production. Batch cultivation is widely used for the confirmation of phenotypes at the lab scale, and for mass production of biomass at the industrial scale. However, the culture conditions change as the cells get older in batch culture, so it can be difficult to determine optimal conditions for production of biomass and target products (Fernandes, Mota, Teixeira, & Vicente, 2015). On the other hand, continuous cultivation allows the maintenance of stable culture conditions. In particular, nutrient concentrations can be maintained by feeding fresh medium continuously using a chemostat, and cell density and photosynthetic efficiency can also be maintained using a turbidostat and luminostat. These various continuous modes allow to maintain optimal growth condition by providing nutrients continuously (Fernandes et al., 2015; Ho et al., 2014). As a result, the productivity of continuous cultivation is generally 2.3‐ to 5‐times higher than batch cultivation systems (Fernandes et al., 2015; Lee et al., 2013). The additional advantages of continuous cultivation are reduced costs for harvesting, labor, cleaning, and sterilization. Continuous cultivation is thus a promising approach for industrial cultivation of microalgae (Fernandes et al., 2015; Mata, Martins, & Caetano, 2010).

We reported previously that the overexpression of NsbHLH2 TF could improve the production of biomass and lipids in *N. salina* (Kang et al., 2015a). However, lipid productivity of NsbHLH2 transformants became similar to that of wild‐type (WT) at the end of the cultivation under the batch culture due to nutrient depletion. In the present study, we thus subjected one of the NsbHLH2 strains to continuous cultivation using a flat‐panel photobioreactor (PBR) for maximized production of biomass and lipids by providing nutrients constantly and maintaining optimal conditions, which was not possible with the batch mode. We then attempted to identify the genes possibly regulated by NsbHLH2 TF that have roles in the growth and lipid synthesis (Anderson, Muff, Georgianna, & Mayfield, 2017), and verified the messenger RNA (mRNA) expression of these genes via quantitative real‐time polymerase chain reaction (qRT‐PCR). Taken together, this study provides proof‐of‐concept that TF engineering with NsbHLH2 can be used for production platform, which can be managed/optimized by continuous cultivation in PBRs.

## 2 MATERIALS AND METHODS

### 2.1 Microalgal strain and continuous cultivation in a flat‐panel PBR

*N. salina* CCMP1776 (National Center for Marine Algae and Microbiota) and *NsbHLH2* overexpressing transformant 3–6 (NsbHLH2 3–6) were maintained in modified f/2, F2N medium, which consists of 15 g l^−1^ sea salt (Sigma‐Aldrich, St. Louis, MO), 10 mM Tris‐HCl (pH 7.6), 427.5 mg l^−1^ NaNO_3_, 30 mg l^−1^ NaH_2_PO_4_∙2H_2_O, 5 ml l^−1^ f/2 trace metal mixture (Kilian, Benemann, Niyogi, & Vick, 2011; Kwon et al., 2018).

A flat‐panel PBR was used for the continuous cultivation of WT and transformant cells (Sung, Lee, Kim, Nam, & Chang, 2017). This PBR has dimensions of 35 mm × 240 mm × 310, and 2.5 l working volume. LED irradiation (100 μmol photons m^−2^ s^−1^) was applied to each side of the PBR, so the total light intensity was 200 μmol photons m^−2^ s^−1^. Microalgal cells were cultivated in the PBR with 0.1 vvm of 2% CO_2_ at 25°C. F2N medium without Tris‐HCl was used for continuous cultivation. For continuous cultivation, F2N medium was supplied using a peristaltic pump (EMS Tech, Yongin‐si, Gyeonggi‐do, South Korea). The working volume was maintained by an overflow channel in the PBR.

### 2.2 Growth and nutrient concentration analysis

The cell growth was assessed by the measurement of the cell density and dry cell weight (DCW). The cell density was determined using a Cellometer Auto X4 Cell Counter (Nexcelom Bioscience, Lawrence, MA). DCW was determined by passing cells through a GF/C filter paper (Whatman, Maidstone, Kent, UK), washing with deionized water, drying at 105°C overnight, and then weighing. The specific growth rate was calculated by the following equation (Bailey & Ollis, 1977; Monod, 1949):

(1) $$\frac{\mathrm{dX}}{\mathrm{dt}}=DX_{f}+\mu X-\mathrm{DX}$$

where, *X*_*f*_ is the biomass concentration of the feed stream, *X* is the biomass concentration in the PBR, *D* is the dilution rate, and *µ* is the specific growth rate. As the feed stream has no biomass, *X*_*f*_ can be ignored. In addition, $\frac{\mathrm{dX}}{\mathrm{dt}}$ is zero when the culture is at steady state. Thus, the above equation can be simplified to:

(2) $$\frac{\mathrm{dX}}{\mathrm{dt}}=\left(\mu -D\right)X=0$$

Therefore, the specific growth at a steady state is the same as the dilution rate. Biomass productivity at steady state was thus calculated by multiplying DCW and dilution rate.

To quantify nitrate (NO_3_^−^) concentration, a supernatant of the microalgal culture. was obtained by filtration through a 0.2 µm syringe filter (Sartorius Stedim Biotech, Gottingen, Germany), and analyzed using ion chromatography (881 compact IC pro, Metrohm, Herisau, Switzerland) with the Metrosep A Supp5 150 column for anions.

We calculated the maximum specific growth rate (*μ*_m_) and the half‐saturation constant (*Ks*) using the Monod equation (Monod, 1949):

(3) $$\mu =D=\frac{\mu_{m}\;\cdot \;S}{K_{S}+S}$$

where, *μ*_m_ is the maximum specific growth rate, *S* is the concentration of the limiting substrate, and *Ks* is the half‐saturation constant. By inverting Equation (3), we obtain the following:

(4) $$\frac{1}{D}=\frac{1}{\mu_{m}}+\frac{K_{S}}{\mu_{m}}\;\cdot \;\frac{1}{S}$$

By plotting of 1/D and 1/S, we estimated *μ*_m_ and *Ks*.

### 2.3 Fatty acid methyl ester analysis

The fatty acid methyl ester (FAME) analysis was conducted as previously described (Kang et al., 2015b). Briefly, a chloroform‐methanol mixture (2:1, v/v) was used for lipid extraction, and samples were then heated to 100°C with methanol and sulfuric acid for transesterification. FAMEs were analyzed by a gas chromatograph (HP 6890, Agilent, Santa Clara, CA) that had a flame ionized detector and an HP‐INNOWax polyethylene glycol column (HP 19091N‐213), and quantified using a 37‐component mix of FAME standards (F.A.M.E. MIX C8‐C24, Sigma‐Aldrich).

### 2.4 Identification and qRT‐PCR analysis of NsbHLH2‐regulated genes affecting growth and lipid synthesis

Target genes that were possibly regulated by NsbHLH2 TF were identified by the TF binding site analysis. E‐boxes, (CAnnTG, where “n” represents any nucleotide), are known as bHLH TF binding sequences (Anderson et al., 2017) and matrix‐scan provided by RSAT was used to identify the sequence patterns (Turatsinze, Thomas‐Chollier, Defrance, & van Helden, 2008). A total of 1261 genes containing E‐boxes in their promoters (500 bp upstream from the start codon of coding sequences [CDS] of each gene) were identified. Gene ontology (GO) annotations of these genes was performed using Blast2GO software (http://www.blast2go.com/; Conesa & Gotz, 2008). The genes containing E‐box in their promoters were compared with the corresponding GO annotations of *N. salina* WT using the WEGO online tool (http://wego.genomics.org.cn/; Supporting Information Figure S1). The Pearson chi‐square test was used for the statistical analysis of the significance (*α* = 0.05). On the basis of GO annotations, 18 genes related to the growth and lipid synthesis were selected to investigate mRNA expression levels. Supporting Information Figure S2 shows the locations of the CDS and E‐boxes in these 18 selected genes.

The expression of mRNA of the selected gene was determined by qRT‐PCR, as previously described (Kang et al., 2017). The cells which are obtained at the mid‐exponential phase were incubated for 3 days in normal or N limitation conditions, and the expression was then measured at Day 0, 1, and 3. The relative expression level was normalized by the housekeeping β‐actin gene. Supporting Information Table S1 shows the primers that were used.

## 3 RESULTS

### 3.1 Effect of dilution rate on cell growth, biomass, and FAME productivities

**Figure 1:**
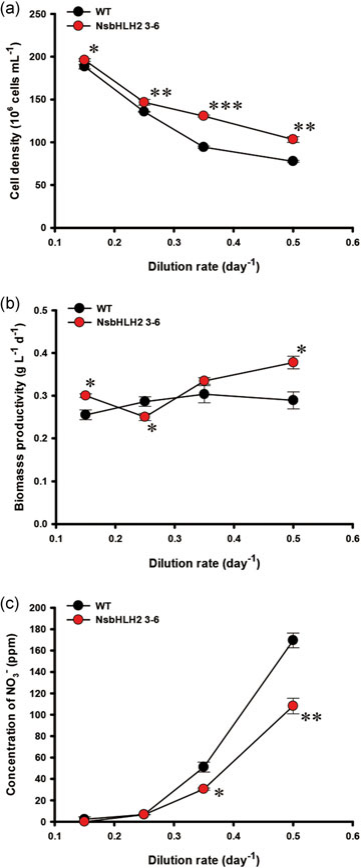
The growth and nutrient concentration analysis of NsbHLH2 transformant according to the dilution rate. (a) Cell density, (b) biomass productivity, and (c) concentration of NO_3_. Feed NaNO_3_ concentration was fixed at 427.5 mg l^−1^. The data points represent the average of samples and error bars indicate standard error (*n* = 3). Significant differences against WT for the same dilution rate conditions, as determined by Student's *t* test, are indicated by asterisks (*p < 0.05, **p < 0.01, ***p < 0.001). NsbHLH2: nannochloropsis salina by overexpressing a basic helix‐loop‐helix transcription factor; WT: wild‐type [Color figure can be viewed at wileyonlinelibrary.com]

In the previous study, we reported the development of two *NsbHLH2* overexpressing transformants, 3–6 and 3–11 (Kang et al., 2015a). We selected the NsbHLH2 3–6 transformant for continuous culture experiments because 3–6 behaved better for the biomass productivity than 3–11 in batch mode experiments. Continuous cultivation was performed using different dilution rates (*D*) with fixed feed NaNO_3_ concentration (427.5 mg l^−1^). As the dilution rate increased, the cell density of the WT and the transformant decreased, but the cell density of the transformant was higher than WT at all dilution rates (Figure 1a). In particular, at high dilution rates of 0.35 and 0.5 day^−1^, the cell density of the transformant was about 38% and 32% higher than that of WT, respectively (Figure 1a). We also assessed the biomass productivity (Figure 1b). At a high dilution rate of 0.5 day^−1^, biomass productivity of the transformant was increased to 0.38 g l^−1^ day^−1^, which was the highest biomass productivity and about 30% higher than that of the WT (Figure 1b). The transformant also consumed more nitrate than WT at high dilution rates of 0.35 and 0.5 day^−1^ (Figure 1c).

**Figure 2:**
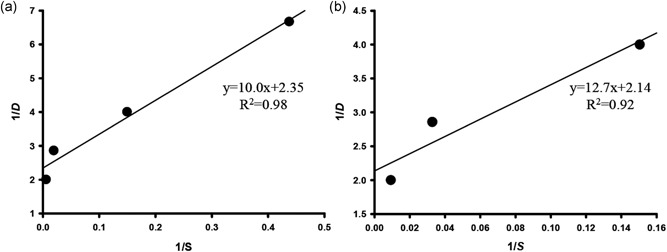
Correlation of 1/*D* and 1/S for the estimation of the maximum specific growth rate (*µ*_m_) and half‐saturation constant (*K*_s_) in the Monod equation [Equation (4)] in the (a) WT and (b) NsbHLH2 transformant. NsbHLH2: Nannochloropsis salina by overexpressing a basic helix‐loop‐helix transcription factor; WT: wild‐type

We determined the maximum specific growth rate (*μ*_m_) and the half‐saturation constant (*Ks*) using the Monod equation (Monod, 1949). A plot of 1/*D versus* 1/*S* with Equation (3) allows calculation of the maximum specific growth rate of the WT (0.43 day^−1^) and transformant (0.47 day^−1^) and of the half‐saturation constant of the WT (5.05 mg l^−1^) and the transformant (5.95 mg l^−1^; Figure 2). Thus, it was confirmed that the transformant had a higher growth rate by consuming more nitrate.

**Figure 3:**
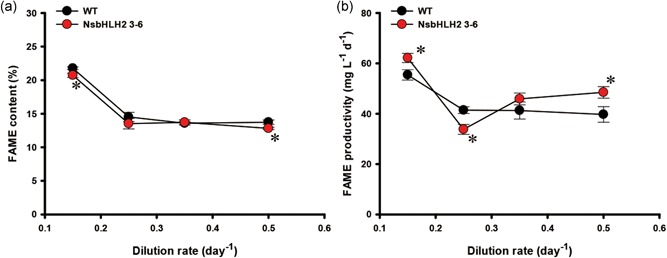
The FAME analysis of NsbHLH2 transformant according to the dilution rate. (a) FAME content and (b) FAME productivity. Feed NaNO_3_ concentration was fixed at 427.5 mg l^−1^. The data points represent the average of samples and error bars indicate standard error (*n* = 3). Significant differences against WT for the same dilution rate conditions, as determined by Student's *t* test, are indicated by asterisks (*p < 0.05, **p < 0.01, ***p < 0.001). FAME: fatty acid methyl ester; NsbHLH2: nannochloropsis salina by overexpressing a basic helix‐loop‐helix transcription factor; WT: wild‐type [Color figure can be viewed at wileyonlinelibrary.com]

We then examined the effect of the dilution rate on FAME production (Figure 3). The total FAME content of WT and the transformant was similar at all dilution rates (Figure 3a). The FAME content of each strain was higher at a lower dilution rate (0.15 day^−1^) than at other dilution rate due to nitrate limitation (Figure 1c). Consequently, FAME productivity of the transformant was increased by 62.2 mg l^−1^ day^−1^ at a dilution rate of 0.15 day^−1^ (Figure 3b).

We also analyzed the effect of the dilution rate on FAME composition (Table 1). The major FAMEs in *N. salina* were palmitic acid (PA; C16:0), palmitoleic acid (POA; 16:1), and eicosapentaenoic acid (EPA; C20:5). The transformant contained lower levels of PA and POA, but a higher level of EPA. As the dilution rate increased, the EPA content increased, and the transformant had the highest EPA content about 33% higher than WT at the dilution rate of 0.5 day^−1^, revealing the potential of NsbHLH2 3–6 as a producer of EPA which is a high‐value product.

**Table 1:** FAME composition of the WT and NsbHLH2 transformant grown in continuous culture at different dilution rates and fixed feed NaNO_3_ concentration of 427.5 mg l^−1^

		FAME composition (%)									
Dilution rate (Day^−1^)	Strain	C14:0	C16:0	C16:1	C18:0	C18:1	C18:2	C18:3	C20:4	C20:5	C others
0.15	WT	6.79 ± 0.02	32.76 ± 0.61	29.55 ± 0.25	1.11 ± 0.01	5.30 ± 0.02	1.17 ± 0.01	0.17 ± 0.17	2.97 ± 0.04	15.58 ± 0.45	4.61 ± 0.30
	NsbHLH2 3–6	6.59 ± 0.02**	32.51 ± 0.15	27.61 ± 0.06***	1.26 ± 0.01***	4.35 ± 0.02***	1.20 ± 0.01*	0.68 ± 0.01*	3.56 ± 0.01***	16.69 ± 0.03	5.55 ± 0.04*
0.25	WT	8.33 ± 0.06	24.16 ± 0.06	26.43 ± 0.03	n.d.	3.48 ± 0.02	1.46 ± 0.01	n.d.	3.75 ± 0.06	25.38 ± 0.05	7.02 ± 0.12
	NsbHLH2 3–6	7.97 ± 0.01**	22.78 ± 0.20**	26.46 ± 0.02	n.d.	1.44 ± 0.03***	n.d.	n.d.	4.17 ± 0.08**	27.97 ± 0.30***	9.21 ± 0.11***
0.35	WT	8.34 ± 0.12	21.18 ± 0.32	24.94 ± 0.32	n.d.	2.80 ± 0.06	1.50 ± 0.02	0.27 ± 0.27	3.82 ± 0.03	30.33 ± 0.18	6.84 ± 0.74
	NsbHLH2 3–6	8.03 ± 0.00	20.39 ± 0.23	24.67 ± 0.04	n.d.	1.76 ± 0.02***	1.01 ± 0.02***	1.00 ± 0.00	3.66 ± 0.04*	31.42 ± 0.22**	8.06 ± 0.13
0.5	WT	8.30 ± 0.03	22.22 ± 0.24	25.49 ± 0.07	n.d.	2.06 ± 0.00	1.15 ± 0.01	0.91 ± 0.03	2.59 ± 0.01	29.18 ± 0.28	7.09 ± 0.24
	NsbHLH2 3–6	7.79 ± 0.01***	20.14 ± 0.20**	24.88 ± 0.07**	n.d.	1.44 ± 0.02***	0.95 ± 0.02***	1.13 ± 0.01**	3.50 ± 0.02*	32.56 ± 0.46**	7.60 ± 0.39

*Note*. FAME: fatty acid methyl ester, NsbHLH2: nannochloropsis salina by overexpressing a basic helix‐loop‐helix transcription factor; WT: wild‐type.

The data points represent the average of samples and error bars that indicate standard error (*n* = 3). Significant differences against WT for the same dilution rate conditions, as determined by Student's *t* test, are indicated by asterisks (*p < 0.05, **p < 0.01, ***p < 0.001).

### 3.2 Effect of feed nitrate concentration on cell growth, biomass, and FAME productivities

**Figure 4:**
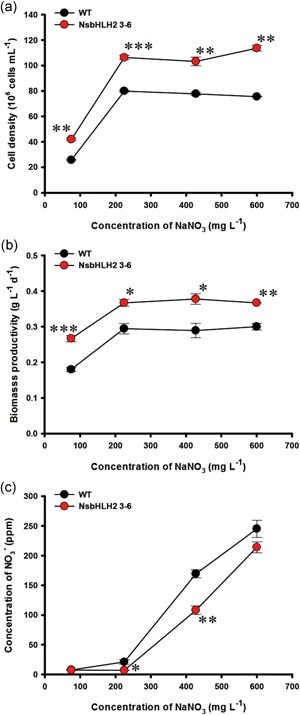
The growth and nutrient concentration analysis of NsbHLH2 transformant according to feed NaNO_3_ concentration. (a) Cell density, (b) biomass productivity, and (c) concentration of NO_3_^−^. The dilution rate was fixed at 0.5 day^−1^. The data at 427.5 mg l^−1^ of feed NaNO_3_ concentration are same as for the dilution rate of 0.5 day^−1^ in Figure 1. The data points represent the average of samples and error bars indicate standard error (*n* = 3). Significant differences against WT for the same feed NaNO_3_ concentration, as determined by Student's *t* test, are indicated by asterisks (*p < 0.05, **p < 0.01, ***p < 0.001). NsbHLH2: nannochloropsis salina by overexpressing a basic helix‐loop‐helix transcription factor; WT: wild‐type [Color figure can be viewed at wileyonlinelibrary.com]

Once we determined the optimum dilution rate of 0.5 day^−1^ for biomass production, we varied another parameter, the feed NaNO_3_ concentration, and tested its effect on the growth and lipid production (Figure 4). Cell density and biomass productivity were higher in the transformant than WT at all tested feed NaNO_3_ concentrations (Figure 4a and b). Cell density and biomass productivity of the transformant and WT were lower at a 75 mg l^−1^ feed NaNO_3_ concentration than at 225 mg l^−1^ or more feed NaNO_3_ concentrations, probably due to the limited nitrate supply. Nitrate uptake was higher in NsbHLH2 than in WT at the feed NaNO_3_ concentration of 225 mg l^−1^ or higher (Figure 4c), consistent with Figure 1c.

**Figure 5:**
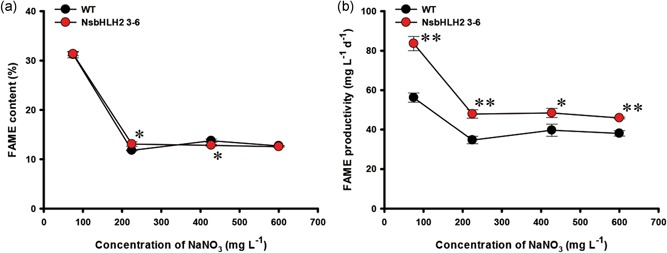
The FAME analysis of NsbHLH2 transformant according to feed NaNO_3_ concentration. (a) FAME content and (b) FAME productivity. The dilution rate was fixed at 0.5 day^−1^. The data at 427.5 mg l^−1^ of feed NaNO_3_ concentration are same as for the dilution rate of 0.5 day^−1^ in Figure 3. The data points represent the average of samples and error bars indicate standard error (*n* = 3). Significant differences against WT for the same feed NaNO_3_ concentration, as determined by Student's *t* test, are indicated by asterisks (*p < 0.05, **p < 0.01, ***p < 0.001). FAME: fatty acid methyl ester; NsbHLH2: nannochloropsis salina by overexpressing a basic helix‐loop‐helix transcription factor; WT: wild‐type [Color figure can be viewed at wileyonlinelibrary.com]

The WT and NsbHLH2 transformant showed similar FAME content under all feed NaNO_3_ conditions (Figure 5a). FAME contents of both WT and the transformant were higher with the 75 mg l^−1^ feed NaNO_3_ concentration compared with those with higher feed NaNO_3_ concentrations (Figure 5a), probably due to nitrogen limitation (Figure 4c). As a result, FAME productivity of NsbHLH2 3–6 reached maximally to 83.6 mg mg l^−1^ day^−1^, 49% higher than that of WT at 75 mg l^−1^ of feed NaNO_3_ concentration (Figure 5b). Overall trends of the FAME composition with different nitrate feed concentrations (Table 2) were similar to those with different dilution rates (Table 1). NsbHLH2 3–6 showed a reduced composition of medium chain FAMEs, while increased polyunsaturated fatty acids (PUFAs) in most nitrate concentrations.

**Table 2:** FAME composition of the WT and NsbHLH2 transformant grown in continuous culture at different feed NaNO_3_ concentrations and a fixed dilution rate of 0.5 day^−1^

NaNO3 conc.		Fatty acid composition (%)									
(mg l^‐1^)	Strain	C14:0	C16:0	C16:1	C18:0	C18:1	C18:2	C18:3	C20:4	C20:5	C others
75	WT	4.71 ± 0.03	45.78 ± 0.18	31.68 ± 0.10	1.32 ± 0.02	3.16 ± 0.13	0.50 ± 0.02	0.73 ± 0.01	1.52 ± 0.01	8.38 ± 0.06	2.20 ± 0.23
	NsbHLH2 3–6	4.05 ± 0.03***	44.10 ± 0.13***	31.63 ± 0.14	1.34 ± 0.00	3.74 ± 0.14*	0.53 ± 0.02	0.76 ± 0.01*	1.86 ± 0.02***	9.16 ± 0.06***	2.83 ± 0.14*
225	WT	9.09 ± 0.04	23.34 ± 0.36	27.16 ± 0.16	n.d.	2.02 ± 0.03	1.18 ± 0.03	n.d.	3.40 ± 0.04	30.89 ± 0.44	2.91 ± 0.05
	NsbHLH2 3–6	8.23 ± 0.22**	25.28 ± 0.83*	26.68 ± 0.14*	n.d.	1.73 ± 0.07**	0.31 ± 0.31*	1.09 ± 0.04	3.62 ± 0.05*	28.94 ± 0.89	4.12 ± 0.08***
427.5^a^	WT	8.30 ± 0.03	22.22 ± 0.24	25.49 ± 0.07	n.d.	2.06 ± 0.00	1.15 ± 0.01	0.91 ± 0.03	2.59 ± 0.01	29.18 ± 0.28	7.09 ± 0.24
	NsbHLH2 3–6	7.79 ± 0.01***	20.14 ± 0.20**	24.88 ± 0.07**	n.d.	1.44 ± 0.02***	0.95 ± 0.02***	1.13 ± 0.01**	3.50 ± 0.02*	32.56 ± 0.46**	7.60 ± 0.39
600	WT	8.32 ± 0.19	22.72 ± 0.42	26.61 ± 0.20	n.d.	2.27 ± 0.00	1.25 ± 0.04	0.94 ± 0.01	3.47 ± 0.08	30.15 ± 0.06	4.28 ± 0.62
	NsbHLH2 3–6	8.21 ± 0.12	22.70 ± 0.51	25.69 ± 0.21*	n.d.	1.53 ± 0.07**	0.95 ± 0.02***	1.08 ± 0.03**	3.64 ± 0.15	31.08 ± 0.27*	5.13 ± 0.56

*Note*. FAME: fatty acid methyl ester; NsbHLH2: nannochloropsis salina by overexpressing a basic helix‐loop‐helix transcription factor; WT: wild‐type.

^a^ These data are same as the data at a dilution rate of 0.5 day^‐1^ in Table 1.

The data points represent the average of samples and error bars indicating standard error (*n* = 3). Significant differences against WT for the same feed NaNO_3_ concentration, as determined by Student's *t* test, are indicated by asterisks (*p < 0.05, **p < 0.01, ***p < 0.001).

### 3.3 Molecular analysis of NsbHLH2‐regualted candidate genes involved in growth and lipid synthesis

**Figure 6:**
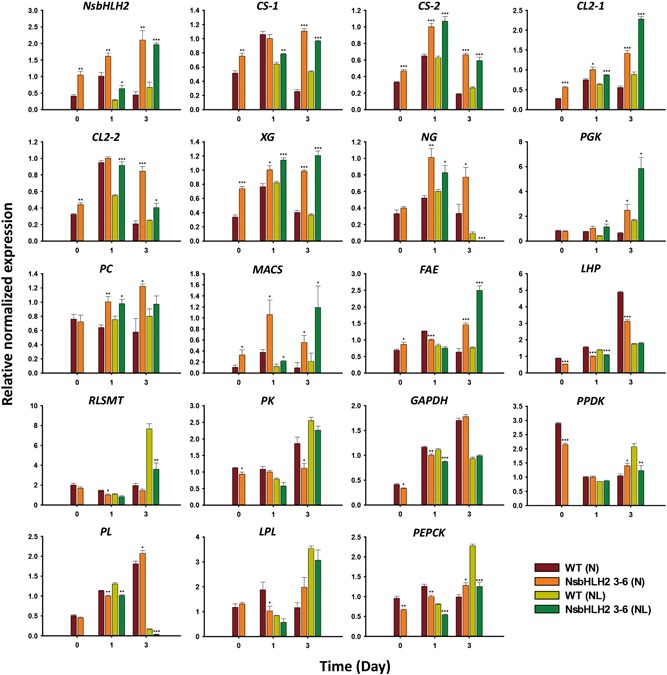
The expression profiles of *NsbHLH2* and NsbHLH2‐regulated genes involved in growth and lipid synthesis in NsbHLH2 transformant. Each mRNA expression was measured at Day 0, 1, and 3 under normal (N) and nitrogen limitation (NL) conditions. The expression of all genes was determined by qRT‐PCR and normalized to β‐actin. Table 3 provides the full names of all the genes. The data points represent the average of samples and error bars indicate standard error (*n* = 3). Significant differences against WT for the same conditions and same time points, as determined by Student's *t* test, are indicated by asterisks (*p < 0.05, **p < 0.01, ***p < 0.001). mRNA: messenger RNA; NsbHLH2: nannochloropsis salina by overexpressing a basic helix‐loop‐helix transcription factor; qRT‐PCR: quantitative real‐time polymerase chain reaction; WT: wild‐type [Color figure can be viewed at wileyonlinelibrary.com]

We identified the genes possibly regulated by NsbHLH2 by screening promoter regions of *N. salina* for the presence of the E‐box (CAnnTG), the binding site of bHLH TFs (Anderson et al., 2017). GO analyses of the identified genes revealed a wide variety of cellular components, molecular functions, and biological processes, including many with unknown functions (Supporting Information Figure S1). Among these, we selected 18 genes that have been known to be related to carbon utilization, glucose and cellulose metabolic processes, photosynthesis, and fatty acid metabolisms (Table 3). These genes and *NsbHLH2* were subjected to qRT‐PCR to determine if their expression is affected by transgenic NsbHLH2 TF, as summarized in Figure 6.

**Table 3:** Growth and lipid synthesis related genes with promoters containing E‐box in *N. salina*

Gene name^a^	Gene ID from *N. salina* CCMP 1776^b^	Homologous gene ID from *N. gaditana* B‐31^c^	Abbreviation	GO Names (ID) list
Cellulose synthase	NSK_05661‐RA	Naga_100049g27	CS‐1	C: membrane; F: cellulose synthase activity; P: UDP‐glucose metabolic process
				F: cyclic‐di‐GMP binding; P: cellulose biosynthetic process
Cellulose synthase	NSK_02572‐RA	Naga_100079g22	CS‐2	C: membrane; F: cellulose synthase (UDP‐forming) activity; P: cellulose biosynthetic process
				P: starch metabolic process; P: sucrose metabolic process; P: UDP‐glucose metabolic process
Cellulase 2	NSK_03190‐RA	Naga_100001g150	CL2–1	P: carbohydrate metabolic process; F: hydrolase activity, hydrolyzing O‐glycosyl compounds
Cellulase 2	NSK_08866‐RB	Naga_100907g1	CL2–2	F: hydrolase activity, hydrolyzing O‐glycosyl compounds; P: carbohydrate metabolic process
Exo‐beta glucanase	NSK_04702‐RA	Naga_100034g4	XG	P: carbohydrate metabolic process; F: hydrolase activity, hydrolyzing O‐glycosyl compounds
Endo‐beta glucanase	NSK_02818‐RA	Naga_100054g30	NG	F: hydrolase activity, hydrolyzing O‐glycosyl compounds; P: carbohydrate metabolic process
Phosphoglycerate kinase	NSK_04201‐RA	Naga_100410g3	PGK	P: glycolysis; F: phosphoglycerate kinase activity; P:gluconeogenesis; P: carbon utilization
				P: phosphorylation
Pyruvate carboxylase	NSK_03518‐RA	Naga_100002g147	PC	F: ATP binding; P: pyruvate metabolic process; P: gluconeogenesis; F: pyruvate carboxylase activity
				F: biotin binding; F: biotin carboxylase activity; F: metal ion binding; P: tricarboxylic acid cycle; P: alanine metabolic process; P: aspartate metabolic process; P: fatty acid biosynthetic process; C: biotin carboxylase complex
Medium chain acyl‐CoA synthetase	NSK_08629‐RA	Naga_100245g4	MACS	F: catalytic activity; P: metabolic process
Fatty acyl elongase	NSK_02920‐RA	Naga_100162g4	FAE	C: integral to membrane
Light‐harvesting protein	NSK_01401‐RA	Naga_100017g83	LHP	P: photosynthesis, light harvesting; C: membrane
Rubisco large subunit methyltransferase, substrate‐binding domain protein	NSK_07857‐RA	Naga_100041g11	RLSMT	n.d.
Pyruvate kinase	NSK_01290‐RA	Naga_100035g36	PK	F: potassium ion binding; F: pyruvate kinase activity; P: glycolysis; F: magnesium ion binding;
				P: gluconeogenesis; P: purine nucleobase metabolic process; P: carbon utilization
Phosphoenolpyruvate carboxykinase	NSK_02536‐RA	Naga_100056g12	PEPCK	P: gluconeogenesis; F: ATP binding; F: phosphoenolpyruvate carboxykinase (ATP) activity;
				P: tricarboxylic acid cycle; P: carbon utilization
Glyceraldehyde‐3‐phosphate dehydrogenase	NSK_08182‐RA	Naga_100081g16	GAPDH	P: oxidation‐reduction process; F: oxidoreductase activity, acting on the aldehyde or oxo group of donors, NAD or NADP as acceptor; F: NADP binding; P: glucose metabolic process; F: NAD binding
Phospholipase like protein	NSK_08630‐RA	Naga_100245g3	PL	n.d.
Lysophospholipase	NSK_08372‐RA	Naga_100156g4	LPL	F: hydrolase activity; P: lipid catabolic process
Pyruvate phosphate dikinase	NSK_07964‐RA	Naga_100043g42	PPDK	F: pyruvate, phosphate dikinase activity; P: pyruvate metabolic process; P:phosphorylation;
				F: ATP binding; F: kinase activity; P: carbon utilization

^a^ Gene name refers to *N. salina* CCMP1776 (https://greenhouse.lanl.gov/greenhouse/) and *N. gaditana* B‐31 (http://www.nannochloropsis.org/index.php) database.

^b^ Gene ID of *N. salina* CCMP 1776 was designated from "Greenhouse" database (https://greenhouse.lanl.gov/greenhouse/).

^c^ Gene ID of *N. gaditana* B‐31 was designated from *N. gaditana* B‐31 database (http://www.nannochloropsis.org/index.php).

We classified the 18 genes based on their expression patterns (upregulation, downregulation, and nonregulation groups) according to culture conditions (normal and N limitation). Under normal condition, the upregulated genes included cellulose synthases (*CS‐1* and *CS‐2*), cellulases (*CL1* and *CL2*), which are involved in cellulose synthesis and degradation, respectively (Shi, Ma, & Lin, 2017). *Nannochloropsis* accumulate chrysolaminarin as carbon reserve which has β‐1,3‐ or β‐1,6‐linked glucose, and it can be degraded to glucose by Exo‐beta glucanase (*XG*) and endo‐beta glucanase (*NG*; Corteggiani Carpinelli et al., 2014; Hildebrand, Manandhar‐Shrestha, & Abbriano, 2017). The NsbHLH2 transformant also showed increased expression levels of the *XG* and *NG* under normal condition. Phosphoglycerate kinase (*PGK*) and pyruvate carboxylase (*PC*) which are involved in Calvin cycle, glycolysis, and gluconeogenesis were also upregulated under normal condition. Two additional upregulated genes included medium‐chain acyl‐CoA synthetase (*MACS*) and fatty acyl elongase (*FAE*) that are known to be involved in fatty acid metabolism (Barker, Larson, Graham, Lynn, & King, 2007). The expression of FAE was increased in the transformant under normal and N limitation condition, which may be correlated to an increased PUFA accumulation (Table 1 and 2).

Downregulated genes included light‐harvesting protein (*LHP*) and Rubisco large subunit methyltransferase (*RLSMT*), which are known to be involved in photosynthesis (Williams & Laurens, 2010). Pyruvate kinase (*PK*), which are involved in glycolysis (Bar‐Even, Flamholz, Noor, & Milo, 2012; Johnson & Alric, 2013), showed downregulation patterns under normal condition.

Certain gene expressions of NsbHLH2 transformant were similar to those of WT, or showed inconsistent patterns under normal condition. Although glyceraldehyde‐3‐phosphate dehydrogenase (*GAPDH*), which is involved in overlapping carbon metabolism including Calvin cycle and gluconeogenesis, was downregulated moderately at Day 1, it showed similar expression levels in NsbHLH2 transformant compared with WT under normal condition (Johnson & Alric, 2013). Similar expression patterns of WT and the transformant were also shown for pyruvate phosphate dikinase (*PPDK*) and phospholipase like protein (*PL*), which are involved in glucose metabolism and lipid catabolism, respectively, under normal condition (Legeret et al., 2016; Radakovits et al., 2012). Lysophospholipase (*LPL*) and phosphoenolpyruvate carboxykinase (*PEPCK*) which are involved in lipid catabolism and gluconeogenesis, respectively (Bar‐Even et al., 2012; Johnson & Alric, 2013), showed inconsistent expression patterns. The expression levels of two genes were lower in NsbHLH2 transformant at Day 1 compared with WT but higher at Day 3 under normal condition (Figure 6).

Under N limitation condition, most genes showed similar expression patterns with those under normal condition, except for only four genes (*PPDK*, *PEPCK*, *PL*, and *LPL*). The four genes of the WT and transformant were categorized as a nonregulation group under normal condition, but the genes showed downregulation patterns in the transformant under the N limitation condition.

Overall, NsbHLH2 appears to positively regulate genes involved in cellulose synthesis and degradation and lipid anabolic processes, while negatively regulated genes were involved in photosynthesis. On the other hand, NsbHLH2 appeared not to consistently regulate the genes involved in glucose metabolism that lie in‐between the carbohydrate and lipid pathways. This may represent activation of carbohydrate and lipid metabolism, resulting in the improvement of the growth and lipid production.

### 3.4 Comparison of cell growth, biomass, and FAME productivities of NsbHLH2 transformant between batch and continuous cultivation

Since performance of NsbHLH2 transformants in batch culture has been reported (Kang, 2015b), we compared our current data of continuous cultivation at a dilution rate of 0.5 day^−1^ to the previous ones (Table 4). Overall, biomass productivity was increased under continuous cultivation, compared with the batch culture. Under normal condition, biomass productivity of WT and NsbHLH2 3–6 was 71% and 114% greater under continuous cultivation than under batch cultivation, respectively. Under N limitation condition, FAME productivity of WT and NsbHLH2 3–6 was 52% and 89% higher under continuous cultivation than under batch cultivation, respectively. Furthermore, under continuous cultivation with normal condition, EPA composition was higher than any other conditions, resulting in the highest EPA productivity in NsbHLH2 transformant. Taken together, continuous cultivation significantly improved biomass, FAME, and EPA productivities of NsbHLH2 transformant, which can be readily applied to scale‐up cultivation for value‐added products and biofuel production.

**Table 4:** Comparison of cell growth and FAME production of the WT and NsbHLH2 transformant under batch culture and continuous culture

		Normal^a^				N limitation^b^			
		Batch^c^		Continuous^d^		Batch^e^		Continuous^d^	
Parameter	Unit	WT	NsbHLH2 3–6	WT	NsbHLH2 3–6	WT	NsbHLH2 3–6	WT	NsbHLH2 3–6
Maximum biomass yield	g L^−1^	2.03 ± 0.12	2.11 ± 0.12	0.58 ± 0.04	0.76 ± 0.03	0.62 ± 0.02	0.77 ± 0.04	0.36 ± 0.01	0.53 ± 0.02
Maximum biomass productivity	mg L^−1^ d^−1^	168.8 ± 10.4	176.0 ± 9.7	288.9 ± 20.0	377.8 ± 14.7	78.1 ± 2.4	96.3 ± 5.4	180 ± 5.8	266.7 ± 8.8
FAME content	% w/w	21.7 ± 0.9	24.1 ± 1.2	13.7 ± 0.3	12.8 ± 0.2	42.3 ± 1.4	46.0 ± 3.3	31.2 ± 0.6	31.3 ± 0.3
FAME yield	mg L^−1^	441.2 ± 37.3	521.2 ± 50.5	79.4 ± 6.2	96.9 ± 4.6	264.9 ± 15.9	352.9 ± 27.6	112.5 ± 4.7	167.3 ± 7.2
FAME productivity	mg L^−1^ d^−1^	36.8 ± 3.1	43.4 ± 4.2	39.7 ± 3.1	48.5 ± 2.3	33.1 ± 2.0	44.1 ± 3.4	56.2 ± 2.3	83.6 ± 3.6
EPA composition	% of total FAME	17.4 ± 1.2	14.5 ± 1.6	29.2 ± 0.3	32.6 ± 0.5	5.9 ± 0.1	4.7 ± 0.3	8.4 ± 0.1	9.2 ± 0.1
EPA yield	mg L^−1^	75.5 ± 3.3	73.3 ± 1.2	23.2 ± 1.7	31.5 ± 1.2	15.5 ± 0.7	16.4 ± 0.7	9.4 ± 0.4	15.3 ± 0.7
EPA productivity	mg L^−1^ d^−1^	6.3 ± 0.3	6.1 ± 0.1	11.6 ± 0.8	15.8 ± 0.6	1.9 ± 2.0	2.1 ± 0.1	4.7 ± 0.2	7.7 ± 0.4

*Note*. NsbHLH2: nannochloropsis salina by overexpressing a basic helix‐loop‐helix transcription factor; WT: wild‐type

^a^ Initial and feed NaNO_3_ concentration in batch and continuous cultivation were 427.5 mg L^−1^ NaNO_3_.

^b^ Initial and feed NaNO_3_ concentration in batch and continuous cultivation were 75 mg L^−1^ NaNO_3_.

^c^ The batch culture of “normal” condition was conducted in Erlenmeyer baffled flasks (200 mL working volume) for 12 days (Kang et al., 2017).

^d^ Dilution rate was 0.5 day^−1^.

^e^ The batch culture of “N limitation” condition was conducted in Erlenmeyer baffled flasks (200 mL working volume) for 8 days (Kang et al., 2017).

## 4 DISCUSSION

*Nannochloropsis* are important industrial microalgae due to their robust growth and high lipid contents, and extensive efforts are dedicated to genetic improvements (Ma, Chen, Yang, Liu, & Chen, 2016; Poliner, Takeuchi, Du, Benning, & Farre, 2018; Radakovits et al., 2012). In particular, TF engineering has been used in *Nannochloropsis* for improving biomass and/or lipid production (Ajjawi et al., 2017; Kang et al., 2017; Kwon et al., 2018); however, these have been achieved in the batch culture in a laboratory scale, and lack assessment of potential for industrial production. We also reported a similar study of NsbHLH2 overexpression in *Nannochloropsis* showing limited improvement in biomass and lipid production under the batch culture (Kang et al., 2015a). We continued improvement of this strain for optimized biomass and lipid production through continuous cultivation by adjusting dilution rate and nitrate concentration in flat‐panel PBR.

We achieved maximal biomass productivity of 377.8 mg l^−1^ day^−1^ in the transformant, 30% higher than WT, at a high dilution rate of 0.5 day^−1^ with a fixed feed of nitrate at 427.5 mg l^−1^. This result is consistent with previous batch culture experiments (Figure 1b). As the dilution rate represents a growth rate at a steady state, as described by Equation (2) (*μ = D*; Bailey & Ollis, 1977; Monod, 1949), the exponential growth phase under batch culture is similar to continuous cultivation at a high dilution rate (0.5 day^−1^). Indeed, the transformant showed high biomass productivity by consuming more nitrate during the exponential phase of batch culture and at a high dilution rate of continuous cultivation (Kang et al., 2015a). In addition, continuous supply of nutrients with the high dilution rate provided a further increased biomass productivity of the transformant in continuous cultivation, 2.1‐fold higher than in batch culture (Table 4).

Nitrogen starvation is the best method for the induction of lipid accumulation (Sharma, Schuhmann, & Schenk, 2012; Zienkiewicz, Du, Ma, Vollheyde, & Benning, 2016). We also improved lipid productivity in NsbHLH2 transformant by providing low feed NaNO_3_ concentration of 75 mg l^−1^ at the fixed high dilution rate (0.5 day^−1^), mimicking the nitrate limitation conditions that we have used in the batch culture (Kang et al., 2015a). As a result, FAME productivity was increased up to 83.6 mg l^−1^ day^−1^, which was about a 90% increase compared with the batch culture. Taken together, we verified that the transformant grew better than WT by consuming more nitrate using continuous cultivation. We could also overcome the limitations of batch culture, including exhaustion of certain nutrients toward the end of the cultivation and accompanied changes of culture conditions. Such nutrient depletion and changes of culture conditions can be overcome by feeding nutrient continuously and maintaining optimal culture condition constantly. This particularly benefited our transformant that consumes nutrient faster than WT, resulting in an increased biomass and lipid production compared with the batch culture.

To understand the mechanism behind the improved biomass and lipid productivities, we identified possible target genes based on the presence of the E‐box that are known to be the binding site for bHLH TFs. Analyses of expression patterns of these genes and their assignment in the central carbon metabolic map revealed interesting patterns as shown in Figure 6 and Supporting Information Figure S3. We confirmed mRNA expression levels of the genes under normal and N limitation conditions, and found that the expression patterns were different according to nitrogen concentration in the medium. Although NsbHLH2 transformant consumed more nitrate compared with WT (Figure 1c and 4c), individual cells of the transformant consumed less nitrate probably due to the high cell density (Supporting Information Figure S4). Overall, the transformant might be under N limitation condition faster than WT. We thus analyzed phenotypes of NsbHLH2 transformant on the basis of mRNA expression patterns with different culture conditions, particularly focused on N limitation condition (Supporting Information Figure S3).

First, positively regulated genes included *CS‐1*, *CS‐2*, *CL2‐1*, and *CL2‐2* that are known to be involved in cellulose biosynthesis and degradation. These contrasting groups of genes may contribute to the growth via enhanced cellulose metabolism. Supporting this possibility, it has been reported that both *CS* and *CL* were highly expressed during the cell division cycle in dinoflagellate (Shi et al., 2017). In addition, overexpression of *CS* genes enhanced cell growth and biomass yield in plants (Hu et al., 2018). It has also been reported that cellulose synthesis and degradation are required for growth and stress response in plants (Kesten, Menna, & Sanchez‐Rodriguez, 2017). Therefore, NsbHLH2 might be able to improve the growth under normal and stress conditions by activated cellulose metabolism via increased expression of *CS*s and *CL*s, consistent with our results showing improved growth and biomass in the NsbHLH2 transformant. Increased *XG* and *NG* expression levels could contribute degradation of chrysolaminarin to glucose (Corteggiani Carpinelli et al., 2014; Hildebrand et al., 2017). As cellulose and chrysolaminarin metabolism lies at the end of gluconeogenesis (Supporting Information Figure S3), upregulation of *XG*, *NG*, *CS*, and *CL* in NsbHLH2 transformant could activate the following downstream carbon metabolism such as glycolysis, TCA cycle, and fatty acid synthesis.

Other carbon metabolic genes that could be regulated by NsbHLH2 included PK, PPKD, and PEPCK, which are involved in the production of pyruvate, phosphoenolpyruvate, and OAA, respectively. Located at the metabolic hub of gluconeogenesis, glycolysis, and TCA cycle, these molecules and enzymes play important roles in carbon concentrating and partitioning depending on cellular needs (Johnson & Alric, 2013; Polle et al., 2014; Radakovits et al., 2012). We observed upregulation of *PC*, and downregulation of *PK*, *PPDK*, and *PEPCK*, which can result in the accumulation of OAA. OAA and associated metabolites may contribute to lipid biosynthesis, in which a cyclic reaction of OAA → malate → pyruvate (catalyzed by malate dehydrogenase, malic enzyme, and PC, respectively) is known to provide nicotinamide adenine dinucleotide phosphate, the critical reducing power for FA synthesis (Ratledge, 2014). It should also be noted that ATP: citrate lyase coverts citrate to OAA by producing acetyl‐CoA that can be used for fatty acid synthesis (Liang & Jiang, 2013; Tan, Lin, Shen, & Lee, 2016). Overlapping with the so‐called pyruvate hub (Dolch et al., 2017), these reactions may contribute to the increased lipid production.

We also observed an increased expression of *FAE* and *MACS* in the NsbHLH2 transformant that can increase long‐chain FAs, while decreased the expression of membrane lipid catabolic genes (*PL* and *LPL*; Legeret et al., 2016; Trentacoste et al., 2013). The outcome of these metabolic regulations might increase accumulation of PUFAs including eicosapentaenoic acid (EPA) (Barker et al., 2007; Kaye et al., 2015), consistent with our FAME profile (Table 1 and 2). Since EPA plays a critical role in thylakoid membrane stability during the growth and stress responses (Hoffmann, Marxen, Schulz, & Vanselow, 2010), its accumulation might positively affect photosynthesis (Legeret et al., 2016). In addition, EPA is one of the value‐added products from microalgae, suggesting potential biotechnological applications for our NsbHLH2 transformants.

The transformant and WT showed almost similar biomass composition when it comes to FAME and carbohydrate contents (Figure 3a, 5a, and Supporting Information Figure S5). Therefore, the aforementioned changed carbon metabolism by NsbHLH2 TF might activate gluconeogenesis, glycolysis, and TCA cycle simultaneously without shifting the flow of carbon biased either way, resulting in an increased growth rate and biomass productivity. In addition, there were negative aspects for the growth such as downregulated photosynthesis genes (*RLSMT* and *LHP*), which can be compensated by positive impacts of metabolic pathways, including activated cellulose and chrysolaminarin metabolism, downstream carbon metabolism, and increased EPA contents.

Taken together, our NsbHLH2 overexpression line could be optimized for maximal production of biomass, lipids, and EPA via continuous cultivation, supported by the regulation of possible target genes that are involved in carbon and lipid metabolism. We previously reported that overexpression of NsbHLH2 can improve biomass and lipid production in *Nannochloropsis* (Kang et al., 2015a), and now provided a solid foundation that this strain can be used in lipid production via continuous cultivation.

## 5 CONCLUSIONS

We examined the use of continuous cultivation in flat‐panel PBR to grow the *NsbHLH2* overexpressing transformant in an effort to ultimately develop a system for the industrial production of biofuels and biomaterials. We confirmed that the transformant showed enhanced biomass, FAME, and EPA production by consuming more nutrients using a continuous cultivation, compared with batch cultivation. In addition, N limitation with continuous cultivation led to the greatest FAME productivity in this transformant. Our examination of the mechanism of the enhanced growth and lipid production in the transformant indicated altered expression of numerous genes, especially those with roles in carbon metabolism. Taken together, NsbHLH2 is expected to be used in mass production of biofuels and EPA from *Nannochloropsis* and other industrial microalgae.

## CONFLICTS OF INTEREST

The authors declare that there are no conflicts of interest.

## Supporting information

Supporting information

## References

[bit26894-bib-0001] Ajjawi, I. , Verruto, J. , Aqui, M. , Soriaga, L. B. , Coppersmith, J. , Kwok, K. , … Moellering, E. R. , others (2017). Lipid production in *Nannochloropsis gaditana* is doubled by decreasing expression of a single transcriptional regulator. Nature Biotechnology, 35(7), 647–652. 10.1038/nbt.3865 28628130

[bit26894-bib-0002] Anderson, M. S. , Muff, T. J. , Georgianna, D. R. , & Mayfield, S. P. (2017). Towards a synthetic nuclear transcription system in green algae: Characterization of *Chlamydomonas reinhardtii* nuclear transcription factors and identification of targeted promoters. Algal Research, 22, 47–55.

[bit26894-bib-0003] Bailey, J. E. , Ollis D. F. (1977). Biochemical engineering fundamentals. New York: McGraw‐Hill.

[bit26894-bib-0004] Bajhaiya, A. K. , Ziehe Moreira, J. , & Pittman, J. K. (2017). Transcriptional engineering of microalgae: prospects for high‐value chemicals. Trends in Biotechnology, 35(2), 95–99. 27387061 10.1016/j.tibtech.2016.06.001

[bit26894-bib-0005] Bajhaiya, A. K. , Dean, A. P. , Zeef, L. A. , Webster, R. E. , & Pittman, J. K. (2016). PSR1 is a global transcriptional regulator of phosphorus deficiency responses and carbon storage metabolism in *Chlamydomonas reinhardtii* . Plant Physiology, 170(3), 1216–1234. 26704642 10.1104/pp.15.01907 PMC4775146

[bit26894-bib-0006] Bar‐Even, A. , Flamholz, A. , Noor, E. , & Milo, R. (2012). Rethinking glycolysis: On the biochemical logic of metabolic pathways. Nature Chemical Biology, 8(6), 509–517. 22596202 10.1038/nchembio.971

[bit26894-bib-0007] Barker, G. C. , Larson, T. R. , Graham, I. A. , Lynn, J. R. , & King, G. J. (2007). Novel insights into seed fatty acid synthesis and modification pathways from genetic diversity and quantitative trait Loci analysis of the *Brassica* C genome. Plant Physiology, 144(4), 1827–1842. 17573542 10.1104/pp.107.096172 PMC1949901

[bit26894-bib-0008] Boyle, N. R. , Page, M. D. , Liu, B. , Blaby, I. K. , Casero, D. , Kropat, J. , … Merchant, S. S. , others (2012). Three acyltransferases and nitrogen‐responsive regulator are implicated in nitrogen starvation‐induced triacylglycerol accumulation in *Chlamydomonas* . The Journal of Biological Chemistry, 287(19), 15811–15825. 22403401 10.1074/jbc.M111.334052 PMC3346115

[bit26894-bib-0009] Conesa, A. , & Götz, S. (2008). Blast2GO: A comprehensive suite for functional analysis in plant genomics. International Journal Of Plant Genomics, 2008, 619832–12. 18483572 10.1155/2008/619832 PMC2375974

[bit26894-bib-0010] Corteggiani Carpinelli, E. , Telatin, A. , Vitulo, N. , Forcato, C. , D’angelo, M. , Schiavon, R. , … Valle, G. (2014). Chromosome scale genome assembly and transcriptome profiling of *Nannochloropsis gaditana* in nitrogen depletion. Molecular Plant, 7(2), 323–335. 23966634 10.1093/mp/sst120

[bit26894-bib-0011] Courchesne, N. M. D. , Parisien, A. , Wang, B. , & Lan, C. Q. (2009). Enhancement of lipid production using biochemical, genetic and transcription factor engineering approaches. Journal of Biotechnology, 141(1‐2), 31–41. 19428728 10.1016/j.jbiotec.2009.02.018

[bit26894-bib-0012] Demırbas, A. (2017). The social, economic, and environmental importance of biofuels in the future. Energy Sources Part B: Economics Planning and Policy, 12(1), 47–55.

[bit26894-bib-0013] Dolch, L. J. , Rak, C. , Perin, G. , Tourcier, G. , Broughton, R. , Leterrier, M. , … Maréchal, E. , et al. (2017). A palmitic acid elongase affects eicosapentaenoic acid and plastidial monogalactosyldiacylglycerol levels in *Nannochloropsis* . Plant Physiology, 173(1), 742–759. 27895203 10.1104/pp.16.01420 PMC5210741

[bit26894-bib-0014] Fernandes, B. D. , Mota, A. , Teixeira, J. A. , & Vicente, A. A. (2015). Continuous cultivation of photosynthetic microorganisms: Approaches, applications and future trends. Biotechnology Advances, 33(6 Pt 2), 1228–1245. 25777495 10.1016/j.biotechadv.2015.03.004

[bit26894-bib-0015] Hildebrand, M. , Manandhar‐Shrestha, K. , & Abbriano, R. (2017). Effects of chrysolaminarin synthase knockdown in the diatom *Thalassiosira pseudonana*: Implications of reduced carbohydrate storage relative to green algae. Algal Research‐Biomass Biofuels and Bioproducts, 23, 66–77.

[bit26894-bib-0016] Ho, S. H. , Ye, X. , Hasunuma, T. , Chang, J. S. , & Kondo, A. (2014). Perspectives on engineering strategies for improving biofuel production from microalgae‐‐a critical review. Biotechnology Advances, 32(8), 1448–1459. 25285758 10.1016/j.biotechadv.2014.09.002

[bit26894-bib-0017] Hoffmann, M. , Marxen, K. , Schulz, R. , & Vanselow, K. H. (2010). TFA and EPA productivities of Nannochloropsis salina influenced by temperature and nitrate stimuli in turbidostatic controlled experiments. Marine Drugs, 8(9), 2526–2545. 20948904 10.3390/md8092526 PMC2953400

[bit26894-bib-0018] Hu, H. , Zhang, R. , Tao, Z. , Li, X. , Li, Y. , Huang, J. , … Peng, L. , et al. (2018). Cellulose synthase mutants distinctively affect cell growth and cell wall integrity for plant biomass production in *Arabidopsis* . Plant and Cell Physiology, 59, 1144–1157. 29514326 10.1093/pcp/pcy050

[bit26894-bib-0019] Ibáñez‐Salazar, A. , Rosales‐Mendoza, S. , Rocha‐Uribe, A. , Ramírez‐Alonso, J. I. , Lara‐Hernández, I. , Hernández‐Torres, A. , … Soria‐Guerra, R. E. , et al. (2014). Over‐expression of Dof‐type transcription factor increases lipid production in *Chlamydomonas reinhardtii* . Journal of Biotechnology, 184(0), 27–38. 24844864 10.1016/j.jbiotec.2014.05.003

[bit26894-bib-0020] Johnson, X. , & Alric, J. (2013). Central carbon metabolism and electron transport in *Chlamydomonas reinhardtii*: Metabolic constraints for carbon partitioning between oil and starch. Eukaryotic Cell, 12(6), 776–793. 23543671 10.1128/EC.00318-12 PMC3675994

[bit26894-bib-0021] Kang, N. K. , Lee, B. , Shin, S. E. , Jeon, S. , Park, M. S. , & Yang, J. W. (2015b). Use of conditioned medium for efficient transformation and cost‐effective cultivation of *Nannochloropsis salina* . Bioresource Technology, 181, 231–237. 25656867 10.1016/j.biortech.2015.01.040

[bit26894-bib-0022] Kang, N. K. , Jeon, S. , Kwon, S. , Koh, H. G. , Shin, S. E. , Lee, B. , … Chang, Y. K. (2015a). Effects of overexpression of a bHLH transcription factor on biomass and lipid production in *Nannochloropsis salina* . Biotechnology for Biofuels, 8(1), 200. 26628914 10.1186/s13068-015-0386-9 PMC4666162

[bit26894-bib-0023] Kang, N. K. , Kim, E. K. , Kim, Y. U. , Lee, B. , Jeong, W. J. , Jeong, B. , & Chang, Y. K. (2017). Increased lipid production by heterologous expression of AtWRI1 transcription factor in *Nannochloropsis salina* . Biotechnology for Biofuels, 10(1), 231. 29046718 10.1186/s13068-017-0919-5 PMC5635583

[bit26894-bib-0024] Kaye, Y. , Grundman, O. , Leu, S. , Zarka, A. , Zorin, B. , Didi‐Cohen, S. , … Boussiba, S. (2015). Metabolic engineering toward enhanced LC‐PUFA biosynthesis in *Nannochloropsis oceanica*: Overexpression of endogenous Δ12 desaturase driven by stress‐inducible promoter leads to enhanced deposition of polyunsaturated fatty acids in TAG. Algal Research, 11, 387–398.

[bit26894-bib-0025] Kesten, C. , Menna, A. , & Sánchez‐Rodríguez, C. (2017). Regulation of cellulose synthesis in response to stress. Current Opinion in Plant Biology, 40, 106–113. 28892802 10.1016/j.pbi.2017.08.010

[bit26894-bib-0026] Kilian, O. , Benemann, C. S. E. , Niyogi, K. K. , & Vick, B. (2011). High‐efficiency homologous recombination in the oil‐producing alga *Nannochloropsis* sp. Proceedings of the National Academy of Sciences of the United States of America, 108(52), 21265–21269. 22123974 10.1073/pnas.1105861108 PMC3248512

[bit26894-bib-0027] Kwon, S. , Kang, N. K. , Koh, H. G. , Shin, S. E. , Lee, B. , Jeong, B. , & Chang, Y. K. (2018). Enhancement of biomass and lipid productivity by overexpression of a bZIP transcription factor in *Nannochloropsis salina* . Biotechnology and Bioengineering, 115(2), 331–340. 28976541 10.1002/bit.26465

[bit26894-bib-0028] Lee, Y.‐K. , Chen, W. , Shen, H. , Han, D. , Li, Y. , Jones, H. D. T. , … Hu, Q. 2013 Basic culturing and analytical measurement techniques. Handbook of Microalgal Culture: John Wiley & Sons, Ltd. 37‐68.

[bit26894-bib-0029] Légeret, B. , Schulz‐Raffelt, M. , Nguyen, H. M. , Auroy, P. , Beisson, F. , Peltier, G. , … Li‐Beisson, Y. (2016). Lipidomic and transcriptomic analyses of *Chlamydomonas reinhardtii* under heat stress unveil a direct route for the conversion of membrane lipids into storage lipids. Plant, Cell & Environment, 39(4), 834–847. 10.1111/pce.12656 26477535

[bit26894-bib-0030] Liang, M. H. , & Jiang, J. G. (2013). Advancing oleaginous microorganisms to produce lipid via metabolic engineering technology. Progress in Lipid Research, 52(4), 395–408. 23685199 10.1016/j.plipres.2013.05.002

[bit26894-bib-0031] Ma, X. N. , Chen, T. P. , Yang, B. , Liu, J. , & Chen, F. (2016). Lipid production from *Nannochloropsis* . Marine Drugs, 14(4), E61. 27023568 10.3390/md14040061 PMC4849066

[bit26894-bib-0032] Mata, T. M. , Martins, A. A. , & Caetano, N. S. (2010). Microalgae for biodiesel production and other applications: A review. Renewable & Sustainable Energy Reviews, 14(1), 217–232.

[bit26894-bib-0033] Monod, J. (1949). The growth of bacterial cultures. Annual Reviews in Microbiology, 3(1), 371–394.

[bit26894-bib-0034] Parmar, A. , Singh, N. K. , Pandey, A. , Gnansounou, E. , & Madamwar, D. (2011). Cyanobacteria and microalgae: A positive prospect for biofuels. Bioresource Technology, 102(22), 10163–10172. 21924898 10.1016/j.biortech.2011.08.030

[bit26894-bib-0035] Poliner, E. , Takeuchi, T. , Du, Z. Y. , Benning, C. , & Farré, E. M. (2018). Nontransgenic marker‐free gene disruption by an episomal CRISPR system in the oleaginous microalga, *Nannochloropsis oceanica* CCMP1779. ACS Synthethic Biolology, 7(4), 962–968. 10.1111/tpj.14314 PMC6616531 29518315

[bit26894-bib-0036] Polle, J. , Neofotis, P. , Huang, A. , Chang, W. , Sury, K. , & Wiech, E. (2014). Carbon partitioning in green algae (chlorophyta) and the enolase enzyme. Metabolites, 4(3), 612–628. 25093929 10.3390/metabo4030612 PMC4192683

[bit26894-bib-0037] Radakovits, R. , Jinkerson, R. E. , Darzins, A. , & Posewitz, M. C. (2010). Genetic engineering of algae for enhanced biofuel production. Eukaryotic Cell, 9(4), 486–501. 20139239 10.1128/EC.00364-09 PMC2863401

[bit26894-bib-0038] Radakovits, R. , Jinkerson, R. E. , Fuerstenberg, S. I. , Tae, H. , Settlage, R. E. , Boore, J. L. , & Posewitz, M. C. (2012). Draft genome sequence and genetic transformation of the oleaginous alga *Nannochloropis gaditana* . Nature Communications, 3, 686. 10.1038/ncomms1688 PMC3293424 22353717

[bit26894-bib-0039] Ratledge, C. (2014). The role of malic enzyme as the provider of NADPH in oleaginous microorganisms: A reappraisal and unsolved problems. Biotechnology Letters, 36(8), 1557–1568. 24752812 10.1007/s10529-014-1532-3

[bit26894-bib-0040] Sharma, K. K. , Schuhmann, H. , & Schenk, P. M. (2012). High lipid induction in microalgae for biodiesel production. Energies, 5(5), 1532–1553.

[bit26894-bib-0041] Shi, X. , Ma, M. , & Lin, S. (2017). Cell cycle‐dependent expression dynamics of G1/S specific cyclin, cellulose synthase and cellulase in the dinoflagellate *Prorocentrum donghaiense* . Frontiers in Microbiology, 8(1118), 1118. 28676796 10.3389/fmicb.2017.01118 PMC5476699

[bit26894-bib-0042] Sung, M. G. , Lee, B. , Kim, C. W. , Nam, K. , & Chang, Y. K. (2017). Enhancement of lipid productivity by adopting multi‐stage continuous cultivation strategy in *Nannochloropsis gaditana* . Bioresource Technology, 229, 20–25. 28092732 10.1016/j.biortech.2016.12.100

[bit26894-bib-0043] Tan, K. W. M. , Lin, H. , Shen, H. , & Lee, Y. K. (2016). Nitrogen‐induced metabolic changes and molecular determinants of carbon allocation in *Dunaliella tertiolecta* . Scientific Reports, 6, 37235. 27849022 10.1038/srep37235 PMC5110973

[bit26894-bib-0044] Trentacoste, E. M. , Shrestha, R. P. , Smith, S. R. , Gle, C. , Hartmann, A. C. , Hildebrand, M. , & Gerwick, W. H. (2013). Metabolic engineering of lipid catabolism increases microalgal lipid accumulation without compromising growth. Proceedings of the National Academy of Sciences of the United States of America, 110(49), 19748–19753. 24248374 10.1073/pnas.1309299110 PMC3856844

[bit26894-bib-0045] Turatsinze, J. V. , Thomas‐Chollier, M. , Defrance, M. , & van Helden, J. (2008). Using RSAT to scan genome sequences for transcription factor binding sites and cis‐regulatory modules. Nature Protocols, 3(10), 1578–1588. 18802439 10.1038/nprot.2008.97

[bit26894-bib-0046] Williams, P. J. L. , & Laurens, L. M. L. (2010). Microalgae as biodiesel & biomass feedstocks: Review & analysis of the biochemistry, energetics & economics. Energy & Environmental Science, 3(5), 554–590.

[bit26894-bib-0047] Zhang, J. , Hao, Q. , Bai, L. , Xu, J. , Yin, W. , Song, L. , … Hu, Z. , others (2014). Overexpression of the soybean transcription factor GmDof4 significantly enhances the lipid content of *Chlorella ellipsoidea* . Biotechnology fo Biofuels, 7(1), 128. 10.1186/s13068-014-0128-4 PMC4159510 25246944

[bit26894-bib-0048] Zienkiewicz, K. , Du, Z. Y. , Ma, W. , Vollheyde, K. , & Benning, C. (2016). Stress‐induced neutral lipid biosynthesis in microalgae ‐ molecular, cellular and physiological insights. Biochimica et Biophysica Acta, 1861(9 Pt B), 1269–1281. 26883557 10.1016/j.bbalip.2016.02.008

