# Supplementary material for: Increased biomass and lipid production by continuous cultivation of Nannochloropsis salina transformant overexpressing a bHLH transcription factor
Source: Biotechnol Bioeng. 2019 Jan 3;116(3):555–68. doi: 10.1002/bit.26894 (PMC6590115; doi:10.1002/bit.26894)
Supplement: Supplementary file 1 — Supporting information [file BIT-116-555-s001.docx]

**Supplementary Information**

**Increased biomass and lipid production by continuous cultivation of *Nannochloropsis salina* transformant overexpressing a bHLH transcription factor**

Nam Kyu Kang^1^, Eun Kyung Kim^1^, Min-Gyu Sung^2^, Young Uk Kim^1^, Byeong-ryool Jeong^2,*^, Yong Keun Chang^1,2,^*

^1^Advanced Biomass R&D Center, 291, Daehak-ro, Yuseong-gu, Daejeon 34141, Republic of Korea

^2^Department of Chemical and Biomolecular Engineering, KAIST, 291, Daehak-ro, Yuseong-gu, Daejeon 34141, Republic of Korea

*Correspondence:

Byeong-ryool Jeong: Department of Chemical and Biomolecular Engineering, KAIST, 291 Daehak-ro, Yuseong-gu, Daejeon 34141, Republic of Korea (+82-42-350-3964; [bjeong@kaist.ac.kr](mailto:bjeong@kaist.ac.kr))

Yong Keun Chang: Department of Chemical and Biomolecular Engineering, and Advanced Biomass R&D Center (ABC), KAIST, 291 Daehak-ro, Yuseong-gu, Daejeon 34141, Republic of Korea (+82-42-350-3927; [changyk@kaist.ac.kr](mailto:changyk@kaist.ac.kr); Fax: +82-42-350-3910)

Running Title: Continuous cultivation of NsbHLH2 transformant

**Supplementary Methods:**

1. Total carbohydrate analysis

Total carbohydrate contents was measured by colorimetric method (Kang et al., 2015). Approximately 3 mg of biomass was vigorously suspended in 10 mL of DW. The 1 mL of suspended biomass samples was mixed with 1 mL of 5% (W/V) phenol solution and 5 mL of sulfuric acid (95~98%). Optical density of the samples were measured at 490 nm using UV/Vis spectrophotometer (UV-1800, Shimadzu, Japan). The contents of total carbohydrate was determined based on standard curves calculated by different glucose concentration solutions.

**Supplementary Figures:**

**
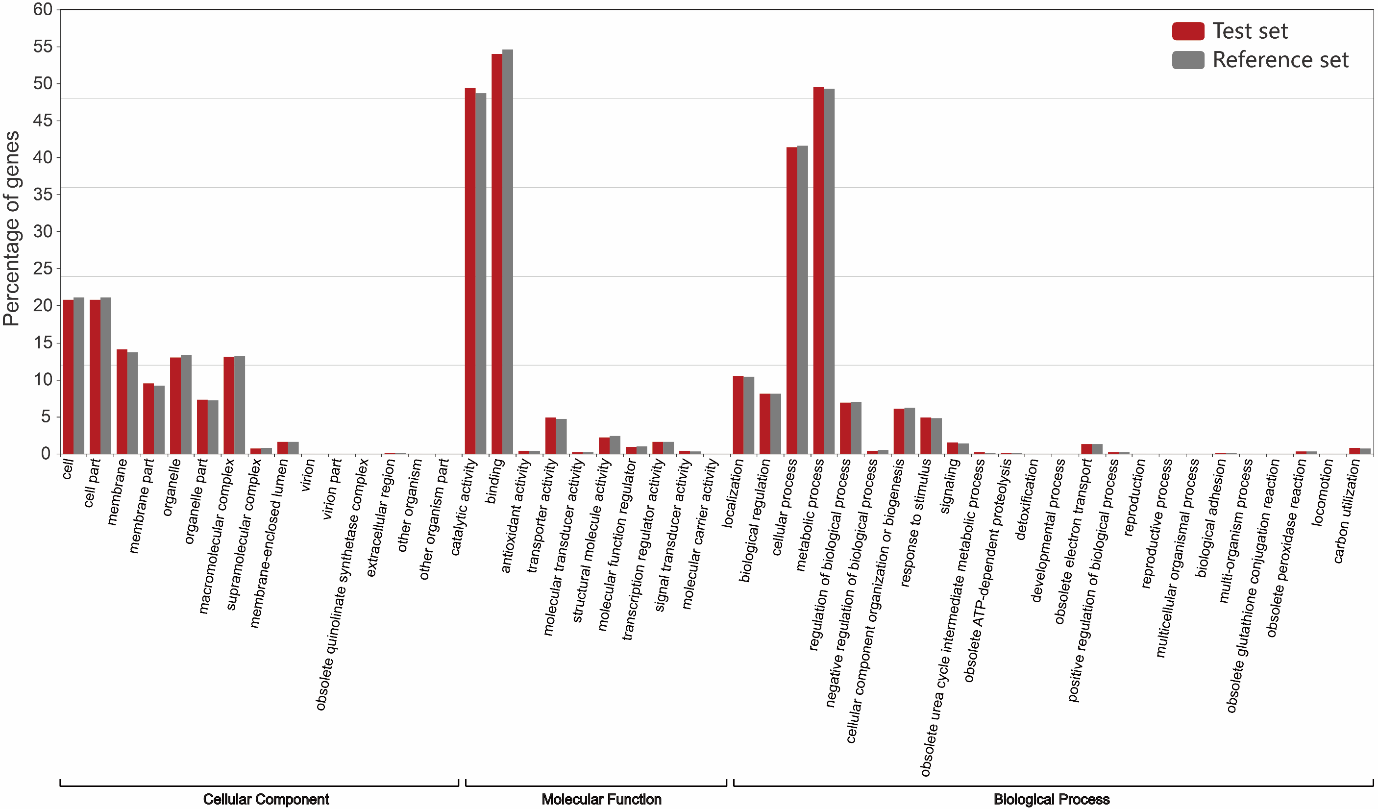
**

**Figure S1.** GO annotation of genes in *N. salina* that have E-boxes in their promoters, based on the WEGO online tool (<http://wego.genomics.org.cn/>). The Pearson chi-square test was employed for statistical analysis, and showed significance (α

=

0.05) Reference set and test set represent *N. salina* whole genes and the genes including E-box in their promoters, respectively.

**
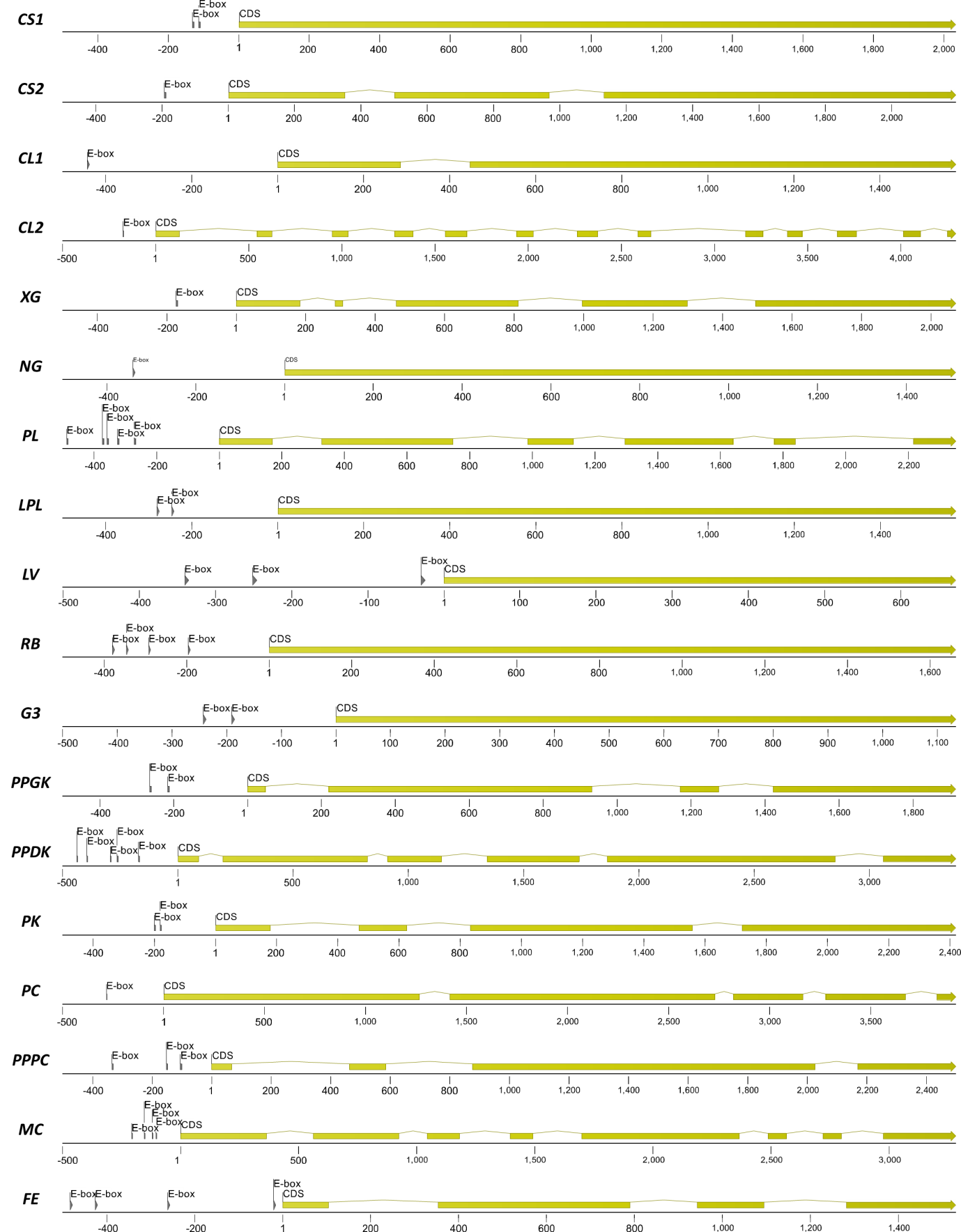
**

**Figure S2.** Schematic of *N. salina* growth and lipid synthesis-related genes containing E-boxes in their promoters*.*

**
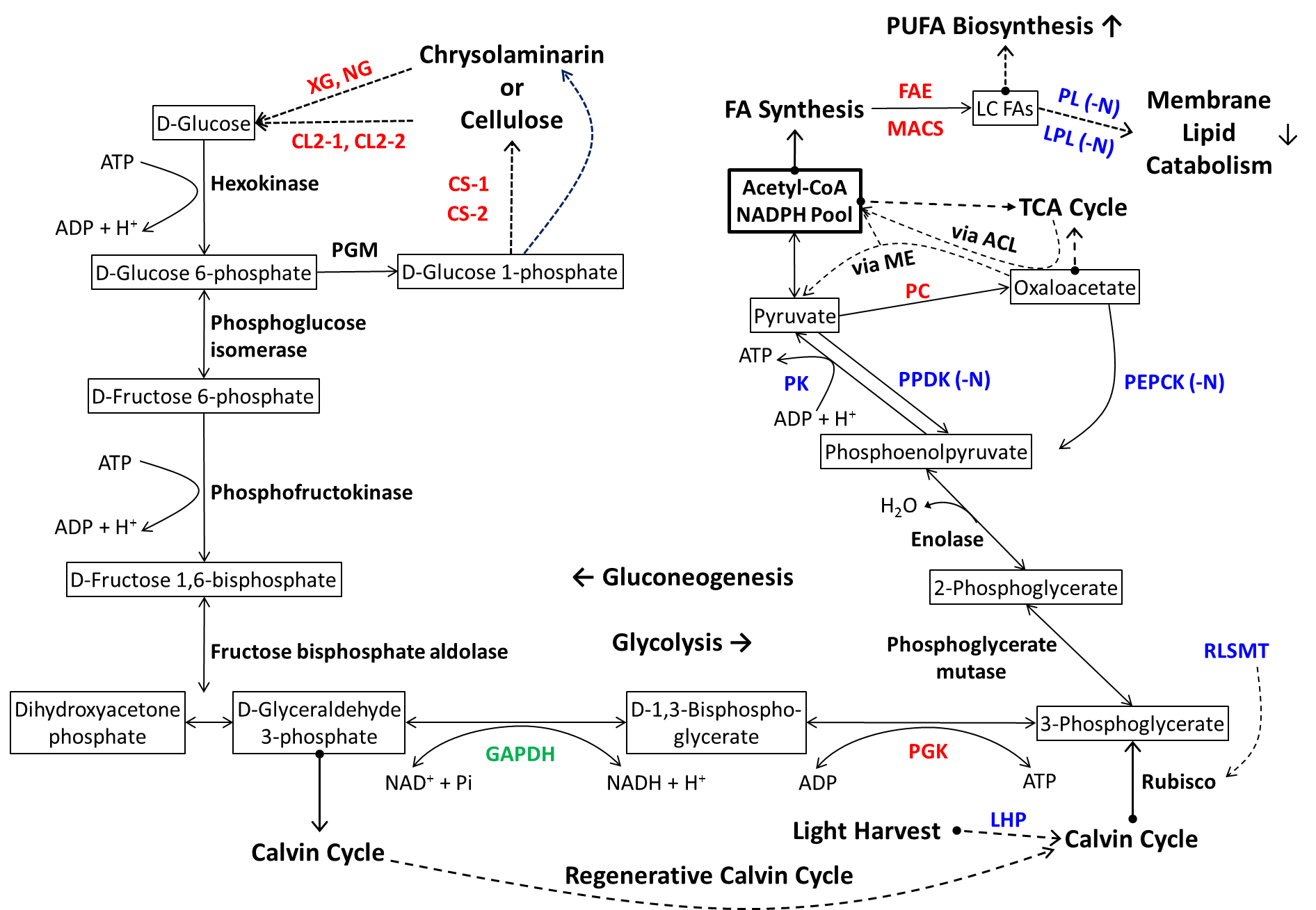
**

**Figure S3.** Simplified metabolic map for possible target genes that might be regulated by NsbHLH2. Mainly shown are glycolysis and gluconeogenesis, together with associated carbon metabolic pathways including cellulose synthesis, Calvin cycle, fatty acids synthesis and membrane lipid catabolism. Solid lines indicate direct or single step reactions, while broken lines indicate indirect or multi-step reactions. Genes analyzed in this study were marked with blue color for down-regulation, red color for up-regulation, and green color for conditional down-regulation. –N means expression levels under N limitation conditions. Overall metabolic pathways, glycolysis and gluconeogenesis in particular, are based on (Bar-Even et al., 2012; Johnson and Alric, 2013). Abbreviations: PGM, phosphoglucomutase; ME, malic enzyme; ACL; ATP: citrate lyase (ACL). Table 3 provides the full names of targeted genes.


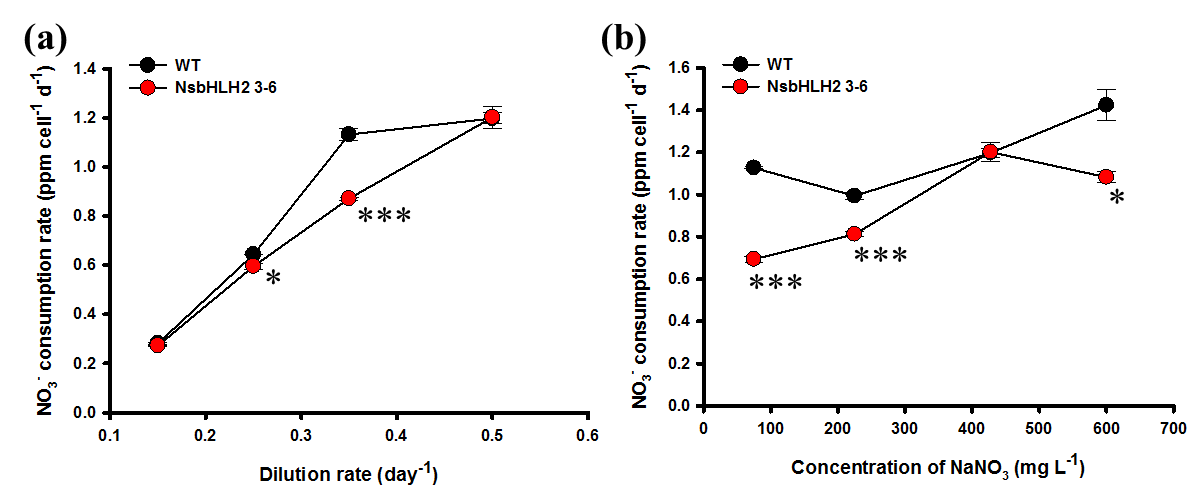


**Figure S4.** Nitrate consumption rate per cell according to (a) dilution rate and (b) feed NaNO_3_ concentration. The data points represent the average of samples and error bars indicate standard error (n=3). Significant differences against WT for the same (a) dilution rate condition and (b) feed NaNO_3_ concentration, as determined by Student’s *t* test, are indicated by asterisks (**P*<0.05, ***P*<0.01, ****P*<0.001).


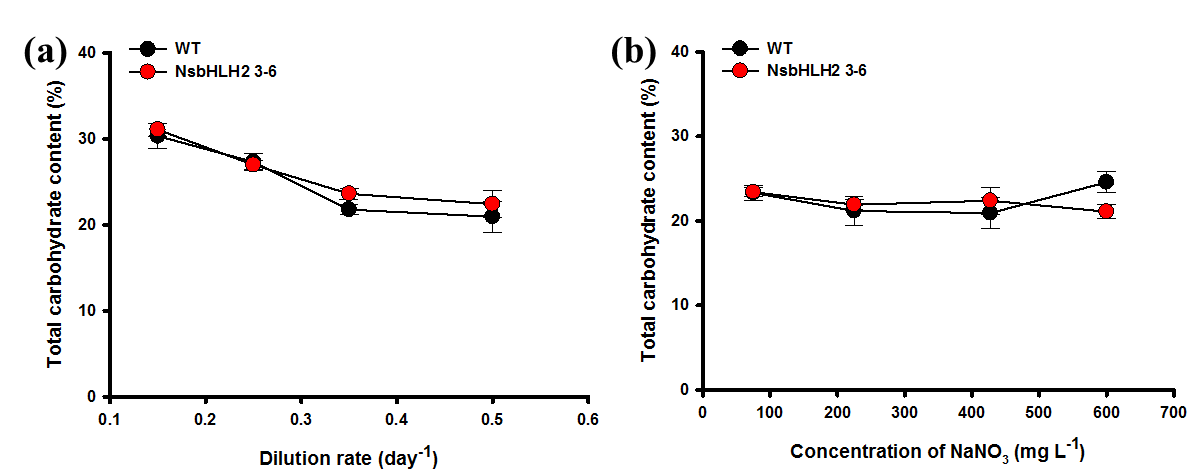


**Figure S5.** Total carbohydrate content according to (a) dilution rate and (b) feed NaNO_3_ concentration. Significant differences against WT for the same (a) dilution rate condition and (b) feed NaNO_3_ concentration, as determined by Student’s *t* test, are indicated by asterisks (**P*<0.05, ***P*<0.01, ****P*<0.001).

**Supplementary Tables:**

**Table S1** Primers used for qRT-PCR.

| Primer | Sequence 5'-3' | Target gene (gene ID)^a^ |
| --- | --- | --- |
| CS-1 fwd | GGCGGATTGGAGATCTTCGT | Cellulose synthase (NSK_05661-RA) |
| CS-1 rev | GTACTGGACCGTGTCGAAGG |  |
| CS-2 fwd | ACGGTCTGGATGCTTTTCGT | Cellulose synthase (NSK_02572-RA) |
| CS-2 rev | AACACCATTTTTCCGTGCCG |  |
| CL2-1 fwd | CCTGCAGGCATGGAGTAACA | Cellulase 2 (NSK_03190-RA) |
| CL2-1 rev | TGTGAATGTGTGCAATGGCG |  |
| CL2-2 fwd | CATCCGGGTTCCCTATTCCG | Cellulase 2 (NSK_08866-RB) |
| CL2-2 rev | TGAAGATTCCTCGCTTCCCG |  |
| XG fwd | CGAAGGTGGGAACGAGTCAA | Exo-beta glucanase (NSK_04702-RA) |
| XG rev | CGGCAGTGCCATTTACGATG |  |
| NG fwd | AGTTTGAGAGCAAACCCCGT | Endo-beta glucanase (NSK_02818-RA) |
| NG rev | AGTAAACTTCCTCGGGTGCG |  |
| PL fwd | GATCGGAGGAGAGCGATTGG | Phospholipase like protein (NSK_08630-RA) |
| PL rev | CTCGGCGATGACAAGGAGTT |  |
| LPL fwd | GACCTGATCGAGACGTGCTT | Lysophospholipase (NSK_08372-RA) |
| LPL rev | ACTCGTCCTCCACGTAGTCA |  |
| LHP fwd | GTTCGTGAAGAGCGGAGACA | Light-harvesting protein (NSK_01401-RA) |
| LHP rev | CGCACGATCAGAACCTTTGC |  |
| RLSMT fwd | CAGGTTTGCCTGGCTGACTA | Rubisco LS methyltransferase,  substrate-binding domain protein (NSK_07857-RA) |
| RLSMT rev | GACGTTTGCGTGGTTGAAGG |  |
| GAPDH fwd | GAGGTCGACTACAAGGGTGC | Glyceraldehyde-3-phosphate dehydrogenase (NSK_08182-RA) |
| GAPDH rev | CACGGTGGTCATAAGTGCCT |  |
| PGK fwd | TGGACGGAAAGACCATCACG | Phosphoglycerate kinase (NSK_04201-RA) |
| PGK rev | CACGAAGGCGACTTTCTTGC |  |
| PPDK fwd | CGACGTGATCACCCTAGACG | Phosphate dikinase (NSK_07964-RA) |
| PPDK rev | CAGTCGGCTGTGTTTGTGTG |  |
| PK fwd | AGCAAGGTAGAAAACGCCGA | Pyruvate kinase (NSK_01290-RA) |
| PK rev | GGCACTTCTACCCCCAAGTC |  |
| PC fwd | GGCAACGTCATCGTCAATGG | Pyruvate carboxylase (NSK_03518-RA) |
| PC rev | CCTTTGTGCTTTCTCACCGC |  |
| PEPCK fwd | CGCCAAGACCATCAACCTCT | Phosphoenolpyruvate carboxykinase (NSK_02536-RA) |
| PEPCK rev | CCGTTTTCGGTTTTGCTCGT |  |
| MACS fwd | GGGCCGTACTACACACCTTG | Medium chain acyl synthetase (NSK_08629-RA) |
| MACS rev | AGGCAGATCCAGTGCTTGAC |  |
| FAE fwd | CTGAAAAACCTGAGCACGCC | Fatty acyl elongase (NSK_02920-RA) |
| FAE rev | GGAGAGCGTGAAAAGGGTCA |  |
| qACTIN fwd | GTGTTTCCCTCCATCGTG | Actin (NSK_01477-RA) |
| qACTIN rev | CCAGTTCGTCACAATACCG |  |

^a^Gene ID of *N. salina* CCMP 1776 was designated from "Greenhouse" database (https://greenhouse.lanl.gov/greenhouse/).

**References**

Bar-Even A., Flamholz A., Noor E., Milo R. (2012). Rethinking glycolysis: on the biochemical logic of metabolic pathways. *Nature Chemical Biology*, *8*(6),509-517.

Johnson X., Alric J. (2013). Central carbon metabolism and electron transport in *Chlamydomonas reinhardtii*: metabolic constraints for carbon partitioning between oil and starch. *Eukaryotic Cell*, *12*(6),776-793.

Kang N. K., Lee B., Shin S. E., Jeon S., Park M. S., Yang J. W. (2015). Use of conditioned medium for efficient transformation and cost-effective cultivation of *Nannochloropsis salina*. *Bioresource Technology*, *181*,231-237.
